# Peer-assisted injection as a harm reduction measure in a supervised consumption service: a qualitative study of client experiences

**DOI:** 10.1186/s12954-020-00455-3

**Published:** 2021-01-06

**Authors:** Em Pijl, Tracy Oosterbroek, Takara Motz, Erin Mason, Keltie Hamilton

**Affiliations:** 1 Faculty of Health Sciences, University of Lethbridge, Lethbridge, Canada; 2 ARCHES, Lethbridge, Canada

**Keywords:** Drug consumption room, Supervised consumption, Injection drug use, Peer assist, Harm reduction

## Abstract

**Background:**

Peer assistance is an emerging area of study in injection drug use. When Canada’s first supervised consumption site (SCS) opened in 2003 in Vancouver, Canada, clients were prohibited from injecting their peers; only recently has this practise been introduced as a harm reduction measure at these sites. In 2018, Health Canada granted federal exemption to allow peer-assisted injection at certain SCS sites, under the Controlled Drugs and Substances Act. Literature pertaining to peer-assisted injection addresses several topics: interpersonal relationships between the injection provider and recipient; the role of pragmatism; trust and expertise; and gender relations.

**Methods:**

In this qualitative study, participants (*n* = 16) were recruited to be interviewed about their experiences in a peer-assisted injection program (PAIP) at one SCS regulated by Health Canada. Interview data were transcribed and thematically analyzed. Quantitative administrative data were used to provide context and to describe the study population, comprised of people in the PAIP (*n* = 248).

**Results:**

PAIP clients made up 17.4% of all SCS clients. PAIP clients were more likely to be female and Indigenous. Injection providers expressed being moved by compassion to help others inject. While their desire to assist was pragmatic, they felt a significant burden of responsibility for the outcomes. Other prominent factors related to the injection provider-recipient relationship were social connection, trust, safety, social capital, and reciprocity. Participants also made suggestions for improving the PAIP which included adding more inhalation rooms so that if someone was unable to inject they could smoke in a safe place instead. Additionally, being required by law to divide drugs outside of the SCS, prior to preparing and using in the site, created unsafe conditions for clients.

**Conclusions:**

Regular use of the SCS, and access to its resources, enabled participants to lower their risk through smoking and to practice lower-risk injections. At the federal level, there is considerable room to advocate for allowing clients to divide drugs safely within the SCS, and to increase capacity for safer alternatives such as inhalation.

Peer-assisted injection, sometimes called ‘doctoring,’ [1] is a common practice among people who inject drugs. Wood et al. found that 22.8% of people who inject drugs (PWID) that were enrolled in the Vancouver Injection Drug User Study (VIDUS) had required help to inject [2]. Some PWID require assistance because they lack experience injecting [3], or on account of physical or psychological limitations [2]. When Canada’s first supervised consumption site (SCS) opened in Vancouver, British Columbia, in 2003, federal law prohibited onsite peer assistance of any kind [4]. This prohibition effectively barred PWID who wished to access the SCS but were unable to self-inject. These individuals were left to inject in hurried, unsafe, and unclean conditions, leading public health leaders and researchers to call on government to allow peer assist within supervised consumption settings [5, 6].

Only recently has peer assistance been introduced at some SCSs in Canada, first through a pilot project granting federal exemption under the Controlled Drugs and Substances Act to a select number of SCSs. As of March 2020, peer assistance has become an official and regulated optional service at SCSs across the country. SCSs are not proactively exempted for peer assistance; they must apply to the Office of Controlled Substances for peer-assistance authorization. Currently, 20 of the 39 operating SCSs in Canada are exempt to offer peer assistance. The present study was one of the first of its kind to examine a federally sanctioned peer-assisted injection program (PAIP), at an SCS in Alberta that participated in the pilot project. Usage of the PAIP, and findings from individual qualitative interviews with the people who assist others to inject and people who are assisted, are reported here. The aim of this qualitative study was to better understand peer-assistance culture and relationships within an SCS.

## Background

Insite, Canada’s first SCS, opened in 2003 in response to high rates of street drug use in the Downtown Eastside of Vancouver, BC. At the time, peer injecting was prohibited on the premises by federal law [4, 7, 8], meaning any clients requiring assistance with injection remained vulnerable to unsafe conditions offsite. In a further effort to mitigate risks such as syringe sharing, blood-borne infection and overdose among this population [3, 9], a Vancouver grassroots organization opened an unsanctioned injection site in the same area, wherein peers oversaw and assisted with injections. Within the literature pertaining to peer-assisted injecting several topics are prominent: interpersonal relationships between the injection provider and recipient; the role of pragmatism; and the role of trust and assessment of expertise.

### Interpersonal relationships

Interpersonal relationships between injection providers and recipients are a key factor in peer-assisted injection. McElrath and Harris [3] described how the recipient and injection provider are often part of a social network, where injections may be reimbursed with cash or drugs. Higher rates of receptive syringe sharing are associated with close social, romantic or intimate relationships, which may arise prior to regular injection assistance between partners, or during its course.

Injection provider–recipient relationships can also be characterized by violence, harm, and inequality. McNeil et al. [10] found that many PWID experience violence when seeking assistance injecting, particularly when those encounters occur in marginal spaces characterized by disorder and risk. Kolla et al. [8] found that women were especially at risk for overdose when receiving injection assistance by a partner. Women are more likely to need help injecting, having smaller veins, and their injection providers are more likely to be male intimate partners or dealers [1, 3]. Such gendered peer injecting tends to produce unequal power relationships, indicative both of gender power differential and the social context of injecting, wherein male injection providers exercise control over female recipients [3]. The commonplace exchange of sex for drugs puts partners doubly at risk, for sexually transmitted disease and blood-borne infections from shared syringes [11]. Women recipients are at much higher risk from unsafe injection practices, as their male injection providers tend to inject themselves first [3]. Wright et al. [12] found that peer injection between intimate, heterosexual couples disproportionately left women vulnerable to physical, economic, and emotional abuse from their male partners. McNeil et al. [13] found that hegemonic masculinity and gendered violence were normative features of drug culture. In relationships characterized by greater trust, egalitarianism and support, peer injecting is more reciprocal [12].

### Pragmatism

While in some cases, educational support can reduce the need for peer assistance [14, 15] peer injections are described as being pragmatically motivated, primarily undertaken because the recipient is experiencing withdrawal, shakiness, and anxiety [3, 16]. Wilkins et al. [17] found that many PWID do not to want to inject others, relenting only due to empathy when they see their friends or partners suffering. In these situations, injection providers perceived their role as ‘helping out.’ Some sites report having an “injection support team” either in unsanctioned sites or as outreach, locating and assisting individuals who require assistance with injecting [16, 18].

### Trust and assessment of expertise

Dechman [19] stated that “natural helpers”—individuals in social, drug using circles whose primary role is secondary distribution of drug use paraphernalia—are frequently requested to inject their peers. Injection providers may be sought out on account of their demonstrated expertise in intravenous injection [3, 19]; some of these individuals have spoken of a “buzz they received from injecting others… assuming the position of the expert injector” [17]. Brothers [1] explores the role of social contracts, technical expertise and trust in the peer-assisted transaction, finding that the degree of trust in the person injecting is assessed in numerous ways. Given that people who assist are considered to have uncredentialed expertise in venipuncture, trustworthiness to perform the task is assessed by non-technical traits including social ties, referrals, or the use of professional rhetoric.

### The public health value of peer assist

There are several reasons that peer-assisted injection in controlled settings like SCSs might be beneficial. From a public health perspective, allowing peer-assisted injection can reduce other risks, since recipients have higher rates of sharing syringes and other paraphernalia [20] and as such, permitting this activity within a supervised facility may provide structured opportunities to enhance the health and safety of people who use drugs. Mitra et al. [21] reported that 32% of potential SCS clients want assisted injection as a standard, operational feature onsite. Peer-based approaches to drug use have proven cost-effective and beneficial to the most vulnerable members of the injection drug using population [22], and peer education is known to be an effective measure against the spread of blood-borne infections [23]. Formalized PAIPs are intended to benefit SCS clients through safe injection education and expert assistance, while protecting recipients from exploitive or unsafe relationships with would be injection providers [16] and the risks associated with sharing equipment and drugs. Public health leaders and researchers have recommended peer assist be a standard part of supervised consumption services in Canada for years [5, 6]. Research continues to emerge to inform the implementation of PAIP on a large scale. In this paper, the terms “injection provider [IP]” and “injection recipient [IR]” refer to individuals delivering and receiving injections, respectively.

## Methods

### Purpose of the study

The purpose of this study was to explore the nature of PAIP at one supervised consumption site in a small city in Alberta. This site was among the first group of SCSs in Canada to receive the Health Canada peer-assist exemption. Through this qualitative study, we sought to understand: (1) the nature of the peer-assist relationship; (2) the experience, attitudes, conditions, and cultures that contribute to and define peer assistance, inside and outside of an SCS; and (3) what features of PAIP design and service delivery can be added, removed, or altered to make the program more user-friendly.

### Study setting

This study explored PAIP usage and client experiences within one of the first government-sanctioned SCS to help inform policy and practice at other SCSs. The city in which this study was conducted is located in traditional Indigenous territory that was colonized by settlers in the 1870s; two First Nations communities are located 63 and 82 km from the city centre. The not-for-profit agency at which this study occurred had provided services to people who have blood-borne infections (such as HIV or HCV) or who use substances, since the mid-eighties. In response to rising rates of opioid use and harms, the agency opened an SCS in late February 2018, and it quickly became one of the busiest sites in North America. It was also the only sanctioned SCS^1^ in North America that provided dedicated space for oral, intranasal and inhalational alternatives to injection.

When the SCS was first open, peer assistance to inject was strictly prohibited by Health Canada. This prohibition meant that clients who needed help to inject could not do so within the SCS and instead, had to use outside of the relative safety of the SCS, putting them at risk for unsafe injections, infections, police interference, and violence. In June 2018, Health Canada exempted six sanctioned SCSs across Canada to allow for peer-assisted injection, as part of the Peer Assistance Pilot Evaluative Framework Project. Over time, the number of sites involved in the pilot expanded to 20, all of which were granted updated exemptions in March 2020 to authorize this service on a long-term basis. The PAIP at this study’s SCS required both injection providers and recipients to sign a participation agreement that stipulated who is authorized by the recipient to provide injections, as well as acknowledging the potential health and legal risks of providing assisted injection. This agreement was kept in client paper files and could be updated as needed to remove or add individuals as their injection provider. Clients were required to sign this onboarding document using their real name; however, after that initial enrolment in the PAIP, clients could continue to use the pseudonym they had selected for when they accessed the SCS. Registered nurses (RN) overseeing peer-assisted injections were responsible for assessing the barrier(s) to self-injection and providing education and support to help the individual work toward independence of self-injection.

The process for requesting peer-assist injection varied client to client and visit to visit. Sometimes, clients identified at reception that they would require assistance and have their injection provider with them. Other times, clients who had several unsuccessful attempts on their own then requested for help from someone they knew can assist and happened to be in the SCS at the time. In every scenario, an RN or licensed practical nurse (LPN) completed an assessment to determine client limitations and assess that the injection provider had the ability to provide assisted injection. Client charts were pulled to confirm participation agreements had been signed by both parties and the procedures and rules of PAIP were understood. The most important rule was that the recipient must inform the injection provider of the type and amount of drug they intended to inject as well as the injection route and site prior to the injection provider injecting the recipient. After overseeing the injection and providing any further education or support, the visit was recorded in the agency’s database neo360^®^ [24]. A spreadsheet captured additional details and observations required for monthly reporting to Health Canada.

### Ethics

Ethical approval was granted by the research ethics office at the academic institution employing the researchers. Research was carried out according to the Tri-Council Guidelines for Human Subjects Research. Participants are identified with pseudonyms throughout this article.

### Data collection

To assess PAIP usage, the researchers were granted access to administrative data gathered by SCS staff members in connection with their duties of assessing, supervising, and reporting onsite peer-assisted injections, in accordance with Health Canada exemption terms and conditions. For each peer-assisted injection, staff recorded a reference code for injection provider and recipient; gender; age; reason for peer assistance; staff interventions; successful/unsuccessful injection; outcome (e.g., overdose); and any open-ended observations. Staff began collecting this data from the outset of the Peer Assistance Pilot Evaluative Framework Project in June 2018.

Participants were recruited through purposive, criterion-i sampling, to ensure inclusion of both recipients and injection providers with a range of peer-assisted use. Operational records from the SCS were scanned to flag a variety of potential participants—female and male; recipient and injection provider; and frequent and infrequent users of peer assistance and supervised consumption. Inclusion criteria for the study were: (1) accessing the SCS during the preceding 10 months; (2) registration in the PAIP through completion of a participation agreement; (3) over the age of 18 years; and (4) identifying either as an injection provider, a recipient, or both. Participants were excluded from the study if they: (1) were not clients of the SCS; (2) had not participated in the PAIP in the preceding 10 months; (3) were under the age of 18; or (4) were acutely intoxicated and/or lacked the cognitive capacity to understand and provide their informed consent.

Potential participants were approached in person, during SCS visits, by a trained research assistant who explained the study and provided a letter of invitation. Those choosing to participate (*n* = 16) gave signed, informed consent prior to taking part in interviews. As with the onboarding documents for the PAIP, clients were required to use their real names when signing the consent form. Of the individuals (*n* = 17) approached for participation in the study, only one individual declined to participate. Of the sample (*n* = 16), there were 9 males and 7 females. Thirteen participants identified as Indigenous, and three were Caucasian. Participants included recipients (*n* = 4), injection providers (*n* = 5), and clients who were both recipient and provider at least once (*n* = 7). The average age of participants was 36.6 years (SD = 10.8, range 18–65 years).

Trained research assistants conducted semi-structured, qualitative interviews in January 2020 in a private room near the SCS reception. Each interview began with demographic questions to identify participants’ various roles (injection provider, recipient, or both). Based on their role, questions were worded to explore participants’ experiences of peer assistance, both onsite in the SCS and offsite in the community; the nature of their relationships with their peers; history of peer assistance; precautions against prevent blood-borne disease; practical concerns; and power differentials within the peer-assistance relationships. All interviews were audio-recorded and professionally transcribed. Each participant received a 25.00 CAD gift card to compensate for their time and contribution to the study.

### Data analysis

Administrative data were used to examine the characteristics of PAIP clients as a whole. These data were analyzed using Chi-square tests and completed with SPSS 26.0.

Interview data were subjected to a thematic analysis [25] conducted by all members of the research team, first independently, then as a team. In the first phase of analysis, each team member became familiar with the data, immersing themselves in the data by reading and re-reading line-by-line the interview transcripts. Following this first phase, as themes emerged from the data, generating initial codes. The team then identified meaningful and salient clusters of codes. The researchers then reviewed these themes, first individually and then as a team, to ensure that the themes captured the salient aspects of the data. Finally, the themes were named using the words of the participants that best reflected the meanings embedded in the data.

## Results

A total of 1,428 unique individuals accessed the SCS between June 12 2018 and December 31, 2019 with 248 unique clients (17.4% of all SCS users) accessing the PAIP. Of these 248 PAIP clients, 94 were injection recipients, 89 were injection providers, and 65 were both recipients and providers on at least one occasion. Just over half of PAIP clients identified as female (*n* = 132, 53.2%), which was significantly higher than the 40.3% (*n* = 575) of SCS clients identifying as female, *χ*^2^(1, *N* = 248) = 17.22, *p* < 0.001). Three quarters (77.8%) of PAIP clients identified as Indigenous (First Nations, Métis or Inuit), which was significantly higher than general SCS clients, of whom 62.2% identified as Indigenous, *χ*^2^(1, *N* = 248) = 25.858, *p* < 0.001).

A Mann–Whitney *U* test was conducted to test whether the average number of visits of PAIP clients (*n* = 248) differed from the general SCS clientele (*n* = 1180) between June 12, 2018 and December 31, 2019. Over that period, PAIP clients had an average of 1,007.98 (*SD* = 1277.44; range 1–9073) visits to the SCS and non-PAIP clients had an average of 87.59 visits (*SD* = 319.71; range 1–4,986). The mean rank of PAIP clients was significantly higher than non-PAIP clients, *U* = 261,990.0, *p* < 0.001. Male PAIP clients were 3.5 times more likely (95% CI 2.735, 4.48) to inject a female recipient than a male recipient, *χ*^2^(2, *N* = 1445) = 104.529, *p* < 0.001.

Overdoses occurring following a peer-assisted injection comprised only 1.02% (*n* = 28) of all site overdoses (*n* = 2758) between June 12, 2018 and December 31, 2019. As is standard procedure at all SCS, all overdoses were reversed.

### Themes

Three themes emerged from the qualitative data: Compassion and pragmatism versus reluctance; safety and risk aversion; and, social connections and the circle of trust. Participants also made suggestions for improving the PAIP. In this section, to indicate the role of the participant, we have provided three signifiers: either IP (injection provider), IR (injection recipient) or IP/R (both roles); the participant’s age; and the participant’s gender (M or F).

#### Compassion and pragmatism versus reluctance

Participants repeatedly expressed compassion for their peers’ suffering, which moved them to help with injections. Participants described not wanting to see others dope sick (experiencing withdrawal); struggling; hurt; “stabbing themselves” [IP/R 34 M]; “wasting their drugs” [IP 30 M]; or causing tissue damage through fruitless and often dangerous attempts to find and then stabilize a vein. Injection providers particularly empathized with those struggling through withdrawal or inability to locate veins, whether on account of skill or physiology, having themselves undergone these tribulations. “I don’t like to see them stab themselves so many times,” remarked one participant, “and when they’re really dope sick, I know how that feels too, so then I’ll give them a hand” [IP 40 M]. Another injection provider was motivated by concern for his peers’ financial circumstances: “I feel bad that they miss every time and they [have] to keep going on. The drugs cost a lot of money, and if they lose [it], they waste it; [and] they’re going to have to try and go get more” [IP 40 M]. Still others provided injections for recipients whom they knew were afraid of needles. Common to all these responses was a willingness to give without recompense, beyond the gratification of providing relief to a fellow PWID in need. As one participant stated: “You get him high, and get him over his [dope] sickness…and I’m happy; he’s happy” [IP/R 54 F].

The injection providers were highly pragmatic about their role, which they saw as unpleasant but necessary. One participant said, about injecting others, “I just don’t like doing it… It’s kind of annoying sometimes…They might OD, so it’s like, that’s always on my mind” [IP/R 34 M]. Some participants acknowledged having acquired reputations among their peers for expertise in venipuncture and owned the injection provider role willingly. The more sought-after injection providers explained they could “doctor them good” [IP 27 F], and find a vein on the first attempt. One participant who needed assistance to inject said she would permit only someone she thought had expertise—as determined by clients and staff—and who she trusted to inject her:

If one of the girls says, ‘I've been injecting so long, ever since they've been here,’ I was like alright. [But] then I kind of got scared so I didn’t really trust them. And then one of the [SCS] workers here [says] ‘She's been injecting a lot of people. I've been watching her.’ [IR 26 F]

Some injection providers felt a strong moral imperative to build recipients’ self-reliance, through teaching and encouragement to gain the skills needed for self-injection. Teaching centered on the recipients’ immediate needs, before and during the time of active assistance. Injection providers actively coached recipients on the techniques of finding a vein and injecting themselves. “I show them, and I tell them, and then I show them,” explained one participant [IP/R 45 M]. “I tell them to watch me when I do it” [IP/R 34 M] and one participant said that the “best way [to learn] is to watch” [IP 40 M]. Skill at peer injecting may enhance self-esteem and provide a sense of purpose: “I feel good about succeeding at what I do,” remarked one participant [IP/R 54 F]. Another participant indicated that she encourages recipients to watch and learn:

I wash my hands, go to them, tie their arm, feel the vein, show them where it is and if they’re comfortable with that part, then I’ll do it and I’ll say – if they don’t want to look at it or … you know, I’ll say, ‘Turn around and look’ [IP/R 43 F].

Attention to detail was frequently emphasized by injection providers, especially for recipients who did not grasp either the inherent risks or the nuances of injection that is acquired over time and practice: “You’ve got to be responsible if you’re going to do this shit, you know …[it’s] dangerous, you know—wrong move, wrong shit—you’ll be gone in a second” [IP/R 43 F]. Some participants described peers gaining the skills over time, reducing their workload as providers: “I haven’t really been injecting anyone lately because everyone pretty much knows how to do it now” [IP/R 24 F].

Smoking in the inhalation room was mentioned by eight participants as a viable alternative to injecting, when an IP could not be found, although the high was described as “different” by several participants and that an injected drug “hits you faster” [IR 32 M]. One participant believed that smoking presented considerable health risks: “I seen like when you smoke a pipe, that thing really gets black like and I wouldn’t want that shit in my lungs [chuckles]” [IP 30 M]. Two participants said they preferred smoking but found the inhalation rooms were often occupied, resulting in a waitlist for the service: “I try and stick to smoking or snorting, but there’s [not enough space back there]…If there was more safe space, I think there’d be more smokers than injectors… people don’t like [to wait]” [IP/R 43 F]. Another participant said “The reason why I started injecting again is because the smoke room is always too hard to get into because of the [waitlist] and so that’s why I just go for an injection and that’s the reason why I started injecting again” [IP/R 49 F]. Reluctant injection providers encouraged would-be recipients to use the SCS inhalation room, where they could smoke their drugs instead: “I just tell them ‘Well if you can’t do it [inject], like why don’t you just smoke it?’” [IP 34 M]. However, two participants indicated that rather than use the inhalation room, if an injection provider was not available, they would leave the SCS to pursue assistance off-site.

Others did not always feel up to the task: “I’ll inject people here, [but] there’s a lot of times I just [don’t] feel like it…I just say no” [IP/R 34 M]. Reluctance to inject others was connected not with selfishness or indifference, but fear of causing harm; injection providers were mindful of missing veins, creating abscesses or other injuries, or wasting recipients’ drugs. Fear of overdosing recipients, however, was paramount. For this reason, many injection providers only injected others in the SCS, where recipients could immediately receive medical treatment. Participants frequently articulated a fear of causing someone to overdose. One participant stated, “I’ve had a few OD on the shot that I’ve given them…Maybe they’ve mixed it wrong… it’s upsetting” [IP 51 F]. This hazard alone was enough to dissuade erstwhile injection providers from continuing the practice, professional supervision at the SCS notwithstanding. “Lately I’ve been trying not to do it for people, because when they OD on it, I’ll feel bad for it” [IP/R 34 M]. Even those injection providers with the skills and knowledge to safely administer a hit actively tried to dodge this responsibility, knowing that failure entailed potentially negative social and emotional consequences: “[The recipient] walks around hating you, because you missed it, and they don’t have no more drugs, or whatever,” explained one participant [IP 40 M]. Another participant was more blunt: “What if they don’t make it through—they die right there? Then I’d feel like it was my fault” [IP/R 19 M].

There was some reluctance among recipients as well, who didn’t always like asking for help to inject. One participant stated that it was very hard to ask for help and “I didn’t like asking my friends [to] do it for me” and that “I'd rather do it myself now” [IR 32 M]. Recipients were grateful to those who would assist and inject them, because they either didn’t know how to inject properly, are “scared to do it by myself” [IR 26 F] or would just “rather get someone else to do it” [IR 26 F]. Recipients sometimes described wanting to learn how to inject themselves, but other times were content to have the help.

#### Safety and risk aversion

Injection providers were scrupulous about procedures and safety, regardless of the recipient or the locale. Much like health care workers adhering to universal precautions, injection providers proceeded on the assumption that everyone was HIV+/HCV+. Participants were asked to describe the steps they go through when injecting someone, and most narratives began with handwashing. Many injection providers wore gloves, provided they did not interfere with locating recipients’ veins: “You know, wash up if you’re at a place, otherwise you could use gloves; I just don’t like handling an injection with gloves” because of the difficulty of finding a vein [IP 51 F]. Several participants indicated that their injection assisting practices at the SCS were not substantially different from their practices off site, wanting to “try and make [recipients] as safe as I can” [IP 51 F].

Recipients prepared their own drugs, which is a program requirement. However, one participant indicated this was likely responsible for an overdose in a client she was assisting: “Maybe they’ve mixed it wrong… They have to mix their own drugs—that’s the-the law here—and then, well obviously they’ll give it to me” [IP 51 F]. Injection providers were required by SCS and PAIP policies to inject recipients prior to injecting themselves, lest they miss veins and cause tissue damage due to intoxication. They also refused to inject peers who were ‘tweaking’ (visibly high on methamphetamine), intoxicated from any substance, unable to sit still, “too needy,” or otherwise at risk. For injection providers, these safety measures were an indicator of both SCS rules and their personal moral compass. “[Even] if I don’t like them, that doesn’t mean I want them to be hurt or anything,” said one participant [IP/R 43 F] with a laugh, “I’m not a hateful person.”

Despite their engagement in what is arguably an intrinsically risky practice (injecting substances), injection providers were fundamentally risk averse. Assisted injections at the SCS, while time consuming because of required protocols, were far preferable to the offsite alternatives, where participants were vulnerable to overdose, contamination, infection, violence, theft, arrest, and social stigma. One participant stated his preference for being assisted in the SCS, because there, “we don’t have to worry about people watching us or the cops pulling in” [IP/R 49 F]. In the participants’ view, SCS safety culture played a significant role in their well-being, particularly when it came to peer assistance, which prior to being permitted was characterized by haste and considerable risk in the outdoor environment. Participants described how the SCS staff would teach safe injection techniques and aftercare, which boosted the knowledge base and self-reliance of injectors, who in turn paid these benefits forward to those they helped. “They’re good people,” one participant said of the SCS nurses who worked in the PAIP [IR 32 M]. One participant described how SCS staff “know what they’re doing, I know that. A few times I needed help and I was really sick…and if I’m gonna do it myself and they tell me, ‘Right here’s your band. Like put it right here…’” [IP/R 54 F].

Due to current Canadian drug laws, drugs must be obtained and divided outside the SCS, which one participant described as logistically challenging because they had to carry out the tasks of drug use in two different places: “I’d get our stuff ready there and then we’ll do our shot here… so then we don’t cut it wherever we’re going” [IP/R 49 F]. Another participant said “sometimes I’m really sick, and then that just takes more time of me having to torture myself, walking over there just to give my friend something, and then coming back” [IP/R 24 F]. Participants pointed out that sharing and dividing drugs in the street effectively negated the protection of the SCS from arrest or self-harm: “It would be better if they just like let us do it here instead of out there because then the cops come and arrest us if they see us or—and then it makes us more angry and want to hurt ourself because we’re so sick” [IP/R 24 F]. One participant said that the PAIP would be better “if we didn’t have to get our drugs offsite or walk away just to give our friend a half of our drug…but all in all it’s pretty good here” [IP/R 24 F].

Additionally, participants found the conditions challenging outside the site: “It’s like a hard time, you know, I never do it outside, because I never get it [the vein], because it’s in the cold… [Then] we just go inside and it’s like no problem” [IP/R 19 M]. Recipients also indicated greater difficulty with being helped adequately “on the street” as opposed to in the SCS and often required more help to inject in that environment [IR 32 M].

#### Social connections and the circle of trust

Of the 16 participants interviewed, only two injection providers acknowledged intimate relationships with their recipients at any time. The rest characterized their peer injection networks as circles of trust, underlain by kinship or kinship-like bonds. These participants saw their peers not just as fellow travelers, but as friends and family. One participant, when asked what their relationships were like with the people they helped, said: “We’re family” [IP/R 19 M]. Several participants described the relationship as being “like family” and at a minimum, friends: “friends, good friends, they’re like family.” [IP/R 27 F].

The emphasis on kinship amongst Indigenous clients—whether by family or band affiliation—contributed to this social order, but street life necessitated unique loyalties and attachments. Familiarity, mutuality, and reciprocity were key factors of the injection provider/recipient dyads. All five injection providers invariably described injecting both males and females, regardless of whether the recipient was the same gender; similarly, the four recipients invariably stated they were injected by both males and females. The seven participants who were both injection providers and recipients were similarly open to either gender, with two of these participants indicating they preferred injecting with their same-gender friends. Only one participant indicated that their current intimate partner injected them.

Injection providers were, however, selective about who they helped, and recipients in turn only sought the assistance of injection providers with whom they had a strong social connection. Similarly, recipients described needing to trust the person who was assisting them, and that trust was earned slowly over time. One participant described a trajectory of trust, from starting out as “I don't really trust anybody around the area” to beginning the peer assist relationship as starting “as friends” [IR 32 M] in the injection provider/recipient relationship.

Injection provider-recipient relationships were characterized by normative, mutual support, and reciprocity. One participant felt it was “common courtesy to give someone a hit” of the drug [IR 48 M]—but also tacitly understood it was purely transactional. Injection providers helped recipients, knowing that certain unspoken benefits would accrue: social capital, goodwill, and respect, as well as tangibles such as gifts of drugs (most commonly mentioned) and other goods. As one participant said, “Sometimes it’s smokes, sometimes it’s drugs” and “sometimes I just do it for free” [IP/R 27 F]. Compensation was a fluid concept; help might be given one day and repaid in a different form on another day. Identity also influenced participants’ attitudes to reciprocity. One Indigenous participant remarked he neither expected nor declined recompense from recipients, because “[in] my culture you can’t deny an offer, or else they’ll take it as [if] I’m dissing them” [IP 30 M].

## Discussion

PAIP clients overall were more frequent SCS users compared to non-PAIP clients, suggesting PAIP is a valuable service for more frequent injection drug users and/or those more willing to access SCS services. Overall, PAIP clients were more likely to be female and Indigenous compared to the general SCS population. From the interviews, gender did not emerge as a prominent factor, which contrasts with the administrative data as well as with the extant literature regarding women who use drugs [26, 27]. It is unclear why these participants indicated they provided and/or received assistance from those of the same and other gender, when the quantitative data indicates there are far more male-to-female assists occurring than female-to-female and male-to-male. The team hypothesized, based on clinical observations, that this may be because some males were well-known injection providers and performed peer assist more frequently, while females were more likely to need assistance.

Relieving the suffering of a fellow human being was the primary motive of the injection providers interviewed [11]. As previously concluded, we found that community bonds, mutuality, and trust within the group were strong [2]. The Indigenous background of the majority of these participants appears to have been a strong factor in forming community bonds, as well as solidarity borne of the isolation and prejudice endured by every participant. Injection provider-recipient relationships were transactional and reciprocal, but participants had flexible attitudes to reciprocity; some recipients reimbursed their injection providers, others paid the favor forward [2], especially in the sharing of knowledge and best practices. While compassion and empathy were the primary drivers of peer assistance, social bonds and trust were equally important prerequisites for each transaction. Among the participants, injection provider-recipient reciprocity amounted to a tacit social contract, based on mutuality and trust rather than power.

Generally speaking, status was not a significant motivator for the injection providers who took part in this study, nor were these individuals treated differently within their peer assistance social circles. While a few participants were respected for their expertise in injecting, they were not venerated as ‘hit doctors,’ in contrast to some other findings [3]. Indeed, participants in our study were comparatively reluctant to engage in peer-assisted injections, owing to a prevalent fear of causing harm. Despite the inherent riskiness of injection drug use [28], participants were largely otherwise risk averse, demonstrating that while the socioeconomic and physical environmental context of drug use can predispose people to risk, actions at the micro-level (such as SCS and PAIP) can significantly lower these risks [29] through offering a safe place for substance use, supportive policies for people who need assistance, and instruction in best practices.

We found no evidence of normalized violence or inequity in the injection provider-recipient dyads investigated, unlike the findings of previous studies [3, 10, 12]. Our data indicated peer assistance was underlain by a spirit of generosity, reciprocity, empathy, and egalitarianism. It is unclear why our sample did not reflect some of the features, such as violence and power imbalances, which are described in the extant literature. It is possible that due to strong kinship ties among the Indigenous community which comprises a large percentage of the SCS’s clients that these cooperative relationships prevailed above a more hierarchical relational model. It is also possible that the program participants are unaware of, or lack the language for, power imbalances within this context, or that they purposefully chose not to disclose violence and imbalances to the interviewers. Alternatively, structural factors such as SCS rules prohibiting violence, or social factors such as the sense of community fostered at the SCS, may account for the lack of violence.

These ‘gender neutral’ findings may be reflective of positive experiences within the SCS setting as compared to studies conducted in community settings, thus supporting the importance of peer assistance availability in SCSs as an important venue to develop safe and positive practices and relationships. However, Kennedy et al. [30] suggest that SCS use is not a protective factor against violence for women, whereas it is associated with lower rates of violence against men. It is also possible that gender-based power imbalances were accepted as the norm (including the SCS as a gendered and racialized space) and that participants do not question that this oppressive structure governs their lives, thus unwittingly [31] or reluctantly [27] participating in it while also suffering under it because of dependence on a partner for basic needs [26].

The injection providers we interviewed demonstrated a highly pragmatic and methodical attitude toward injection. Despite the seeming paradox of taking precautions for an inherently harmful practice, safety measures seemed to represent a locus of control for participants, who otherwise felt powerless to influence the course of their substance use. Every client had the capacity to wash their hands, wear gloves, and observe universal precautions on blood contamination. Even when injecting themselves and others offsite, clients described following the same protocols they learned at the SCS, where these steps were mandatory. This finding is significant, as SCSs are often associated with short-term prophylactic benefits such as preventing overdoses, injuries, infections, and clinical interventions. That regular usage of an SCS changes the context for healthier choices, and promotes safer behaviors among PWID even away from the SCS, has also been suggested elsewhere [14, 15].

It is unclear from this data the degree to which these clients, versus the SCS program, introduced the culture that exists within the program. While the participants described participating in a culture of injection safety, the researchers did not observe the participants’ environment, resources and behavior either at the SCS or offsite, and it is unclear whether the Hawthorne effect contributed to the overwhelmingly positive reports. Because the SCS, as a fixed location, is only one aspect of harm reduction for people who use drugs, advocates are developing programs that reach further into populations. One such initiative is injection support teams, which use peer outreach workers to provide education, instruction and assistance for people who inject drugs, particularly in public places [6, 16, 18, 22, 33]. Such outreach programs can effectively bring peer assistance to the people who require this help, without the restrictions of a formal supervised consumption service.

Overall, PAIP clients were more frequent SCS users, suggesting PAIP is a valuable added service for more frequent injection drug users and/or those more willing to access SCS services. Those who were both providers and recipients were more likely to use the PAIP program than those who were only a provider or recipient alone, suggesting that lack of injection experience or permanent physical limitations were not the primary motivator for PAIP participation [2].

Notwithstanding these encouraging trends, it was also apparent the PAIP and other onsite resources did not go far enough in reducing some participants’ risks. The necessity of dividing and sharing drugs offsite—owing to a technicality in Canadian drug trafficking law which defines any transfer of drugs between individuals as trafficking [32]—still left participants vulnerable to violence, theft, or arrest on the street, and some participants suggested the nuisance of leaving and returning outweighed the benefits of injecting with professional supervision. This application of the law, reviled as arbitrary and unfair, created an unwelcome hurdle in the peer assistance process and a barrier to service delivery. Likewise, many participants—injection providers and recipients—viewed supervised inhalation as a much preferable and safer alternative, only to be frustrated by the limited onsite capacity for this practice. These deficits highlight the importance of timely and responsive knowledge translation in harm reduction research.

### Limitations

The uniqueness of local culture—Indigenous beliefs and value systems in particular—may limit transferability of the study findings to other research settings. The Hawthorne effect may explain why participants described overwhelmingly positive experiences with peer assist. Administrative data were used to quantify individual-level visit numbers; however, this measure does not explain the visit frequency patterns.

## Conclusions

In this small sample of PWID, in a relatively small population center in Alberta, peer assistance culture was found to manifest values of empathy, compassion, trust, protectiveness, and solidarity. That these values were equally connected with street life and First Nations traditions—also subject to widespread prejudice—shows the extent to which the battle for harm reduction and basic human dignity is a battle of perception. The PAIP was found to be a valuable service for more frequent injection drug users and/or those more willing to access SCS services. Greater availability of the supervised inhalation rooms was identified by participants as a way to further reduce the risks associated with substance use. Additional safety measures, such as the redefinition of criminal code restrictions on trafficking and greater availability of supervised inhalation, will come only through advocacy informed by frontline data. This study illustrates one research pathway to accelerate the pace of reform in Canadian controlled substance policy and public awareness.

## Data Availability

We are not able to share our research data publicly, because individual privacy could be compromised and because the participants are part of a marginalized and stigmatized group requiring protection.

## Abbreviations

HIV: Human immunodeficiency virus
HCV: Hepatitis C virus
IP: Injection provider
IR: Injection recipient
OD: Overdose
PAIP: Peer-Assisted Injection Program
PWID: People who inject drugs
SCS: Supervised consumption service

## Footnotes

1 There are some Opioid Prevention Sites (OPS) who offer inhalation services. As of this writing, this was the only SCS with indoor ventilated inhalation rooms.
